# Nonoxynol-9 berberine plural gel has little effect on expression of SLPI, SP-D and lactoferrin in mice’s vagina

**Published:** 2013-07

**Authors:** Qiao Yuan, Chen Zhuo, Ma Yonggui, Lu Fuer, Chen Suhua, Huang Guangying

**Affiliations:** 1 *Department of Integrated Traditional Chinese and Western Medicine, Tongji Hospital. Tongji Medical College, Huazhong University of Science and Technology, Wuhan, P.R.China. *; 2 *Institute of Integrated Traditional Chinese and Western Medicine,Tongji Medical College, Huazhong University of Science and Technology, Wuhan, P.R.China.*; 3 *Department of Obstetrics and Gynecology, Zhongnan Hosptial of Wuhan University, Wuhan, P.R.China.*; 4 *Department of Pharmacy, Tongji Hospital, Tongji Medical College, Huazhong University of Science and Technology, Wuhan, P.R..China.*

**Keywords:** *Vagina*, *Anti-infective agents*, *Mice*, *Berberine*, *Nonoxynol-9*

## Abstract

**Background:** The most frequently used spermicide Nonoxynol-9 (N-9) in the clinic alters the vaginal flora, which will result in an increased risk of opportunistic infection. So development of a novel spermicidal and microbicidal drug appears to be inevitable. Vaginal local immune is an important part of vaginal flora. Secretory leukocyte protease inhibitor (SLPI), surfactant proteins D (SP-D), and lactoferrin (LF) are anti-microbial molecules with important roles in immune system of female vaginas.

**Objective:** To observe effect of a vaginal spermicide nonoxynol-9 (N-9) berberine plural gel on the expression of SLPI SP-D and LF in mice’s vaginas.

**Materials and Methods** **:** Female BABL/C mice were randomly divided into following 5 groups: normal control group, blank gel group, berberine gel group, 12% N-9 gel group and N-9 berberine plural gel group. Estradiol benzoate at physiological dose was done by hypodermic injection to every group’s mice. After 72h, drug gels were separately injected into the mice’s vaginas, while immunohistochemistry and Western blot were taken to detect the expression of the 3 indexes in mice’s vaginas respectively after 24h and 72h of gel injection.

**Results:** The differences in the three indexes between normal control group and blank gel group were not significant statistically (p>0.05). The expression of the three indexes in 12% N-9 gel group was decreased compared to that in blank gel group (p<0.05). The differences in the three indexes between N-9 berberine plural gel group and blank gel group were not significant statistically (p>0.05). Also, the three index's level of 24h and 72h in sub observation groups after treatment were without statistical significance (p>0.05).

**Conclusion:** Application of N-9 berberine plural gel had little impact on antimicrobial peptides in normal mice’s vaginas.

## Introduction

Reproductive tract infections (RTIs) were a serious public health problem in most parts of the world. The World Health Organization (WHO) reckoned that each day one million people acquired a sexually transmitted infection/ RTI, which has reached epidemic proportions throughout the world (1). Failure to combating RTIs at early stage may result in severe complications, including endometritis, uterine sinechiae, salpingitis, pelvic inflammatory disease, as well as female infertility. Topical contraceptives were adopted widely by women with child-bearing age, because of their excellent features, such as reversibility, voluntary selectivity. As the most common topical contraceptive, Nonoxynol-9 (N-9) may fight against some pathogens of RTIs, but it both stimulated vaginal mucosa and broke the balance of vaginal normal flora at the same time, which limited its use. Vaginal agents with dual function of prevention of RTIs and contraception were gradually focused by researchers (2-6). 

Our pre-clinical research found that a new compound N-9 berberine plural gel not only places good synergism of spermicidal, contraceptive effect and bacteriostatic, bactericidal activity, but reduce side effects of N-9 (7-11). Female reproductive tract (FRT) was a unique immunological site that is required to protect the mucosa form a variety of pathogens. A number of broad-spectrum antimicrobial peptides presented in it prevented and reduced infection by directly interfering with the infectivity of the pathogen or indirectly recruiting innate and adaptive immune cells to FRT (12). Imitating the clinical application of it, N-9 berberine plural gel was applied for one time to acquire the influence on antimicrobial peptides of mice’s vaginal flora, including secretory leukocyte protease inhibitor (SLPI), lactoferrin (LF) and surfactant protein D (SP-D). Therefore, the specific objective of this study was to evaluate effects of N-9 berberine plural gel on antimicrobial peptides of mice’s vaginal flora, including secretory leukocyte protease inhibitor (SLPI), lactoferrin (LF) and surfactant protein D (SP-D) when applied vaginally for one time.

## Materials and methods

**Drug and main reagent**

Carbomer was taken as stroma and added into berberine or N-9 berberine in a certain proportion, so as to prepare drug gel; blank gel was also prepared with carbomer as stroma in the same proportion. The five gels above were provided by the Department of Pharmacy of Tongji Medical College of Huangzhong University of Science and Technology (Hubei, China); pH=5.0 of all gels was adjusted by NaOH. Estradiol Benzoate (E_2_) injection（1mg/ml，KingYork Amino Acid Co., Ltd); rabbit anti-SLPI, goat anti-LF polyclonal antibody, mouse anti-SP-D monoclonal antibody, GAPDH antibody and β-actin antibody were purchased from Santa Cruz Company; protein Marker (Fermentas Company American); SP Immunohistochemistry kit and DAB color kit was bought from Zhongshan Goldenbridge Biotechnology Limited Company.

**Animals and grouping**

Animal ethical approval was obtained for the experiments reported in this study: approved by Experimental Animal Center of Tongji Medical College of Huazhong University of Science and Technology-Approval number 00000485 and by the Tongji hospital, Tongji Medical College, Huazhong University of Science and Technology Ethics Committee- Approval number 2012-1203. 

100 female BABL/C mice were randomly divided into five groups, with 20 mice in each group. That is, normal control group, blank gel group (stroma control group) (0.3mg/ml), 12% N-9 gel group , berberine gel group and N-9 berberine plural gel group (0.3mg/ml, 50mg berberine +10%N-9), while each group was divided into two sub observation groups, which were 24h and 72h after treatment. Animals were housed in a temperture- and light- controlled room with 12 h light/dark intervals, with free access to food and water.

**Before** **administration** **, each mouse was treated with the entry of oestrus**

Before 72h of administration, each mouse was injected with Estradiol Benzoate (E_2_) as pretreatment. E_2_ was indicated every other day until the end of the experiment (13). In 24h observation group after treatment, groups were injected E_2_ on the first day of the experiment, and once on the third and the fifth day. On the fourth day, vaginal administration was conducted, after 24h of which, the mice were killed by CO_2_ asphyxiation for sampling; in 72h observation group after treatment, groups were injected E_2_ on the first, third, fifth and seventh day. Vaginal administration was conducted on the fourth day of injection, after 72h of which, the mice were killed by the same method.

**Mice’s vaginal administration**

In all observation groups, 20μL corresponding drug gel was indicated intravaginally for one time. The mice were upside down, so as to make sure that the drug was completely contacted with vaginal mucosa. In normal control group, normal saline at the same amount was injected into mice’s vaginas, and the same operation was done. 

**Hematoxylin eosin (HE) staining**

A part of vaginal slice in all observation groups were being HE stained. The variations of the pathology in mice vaginas were observed under an optical microscope.

**Immunohistochemistry**

Slices of mice vaginas from all observation groups were taken out to determine SLPI and SP-D, LF positive expression by immunohistochemistry SP method, and the method explained by Kumar *et al* (14). PBS was used as a negative control instead of primary antibodies. The first antibodies were rabbit anti- SLPI or goat anti-LF polyclonal antibody, mouse anti-SP-D monoclonal antibody (at a dilution of 1:1000). Under Nikon optical microscope, positive signals were presented as yellowish-brown or brown particles. The image analysis was conducted by Image Pro Plus 5.0, so as to determine positive material expression areas and integral optical density.

**Western blot** **detection**

Referring to the instruction of kits, vaginal tissues of each group’s mice were taken and added into tissue lysate. Bradford method was used to measure protein concentration. In per sample, 50μg 10% or 15% of SDS-PAGE were taken and wet electro-transferred to Nitrocellulose membrane. The membrane was sealed into 5% skimmed milk powder for 2h at room temperature, and then added into primary antibodies at a dilution 1:1000. It was left overnight at 4^o^C. 

After washing the membrane, goat anti-rabbit second antibody or monkey anti-goat second antibody marked with HRP was added(diluted according to 1: 5000), being incubated for 2h at room temperature. It was developed and exposed by adding ECL after washing the membrane, and film was developed. In order to calculate the relative content value of SLPI, LF and SP-D，integral value of protein banding′s optical density was analyzed by American Bio-Rad Quantity One. 

**Statistical analysis**

All the experiments were repeated 3 times. SPSS 17.0 statistical software was adapted to process data, while all experimental measured values were displayed by mean±SD. The data was subjected to one-way ANOVA test, while p<0.05 was considered to be significant.

## Results

**Vagina HE staining**

**Figure 1:**
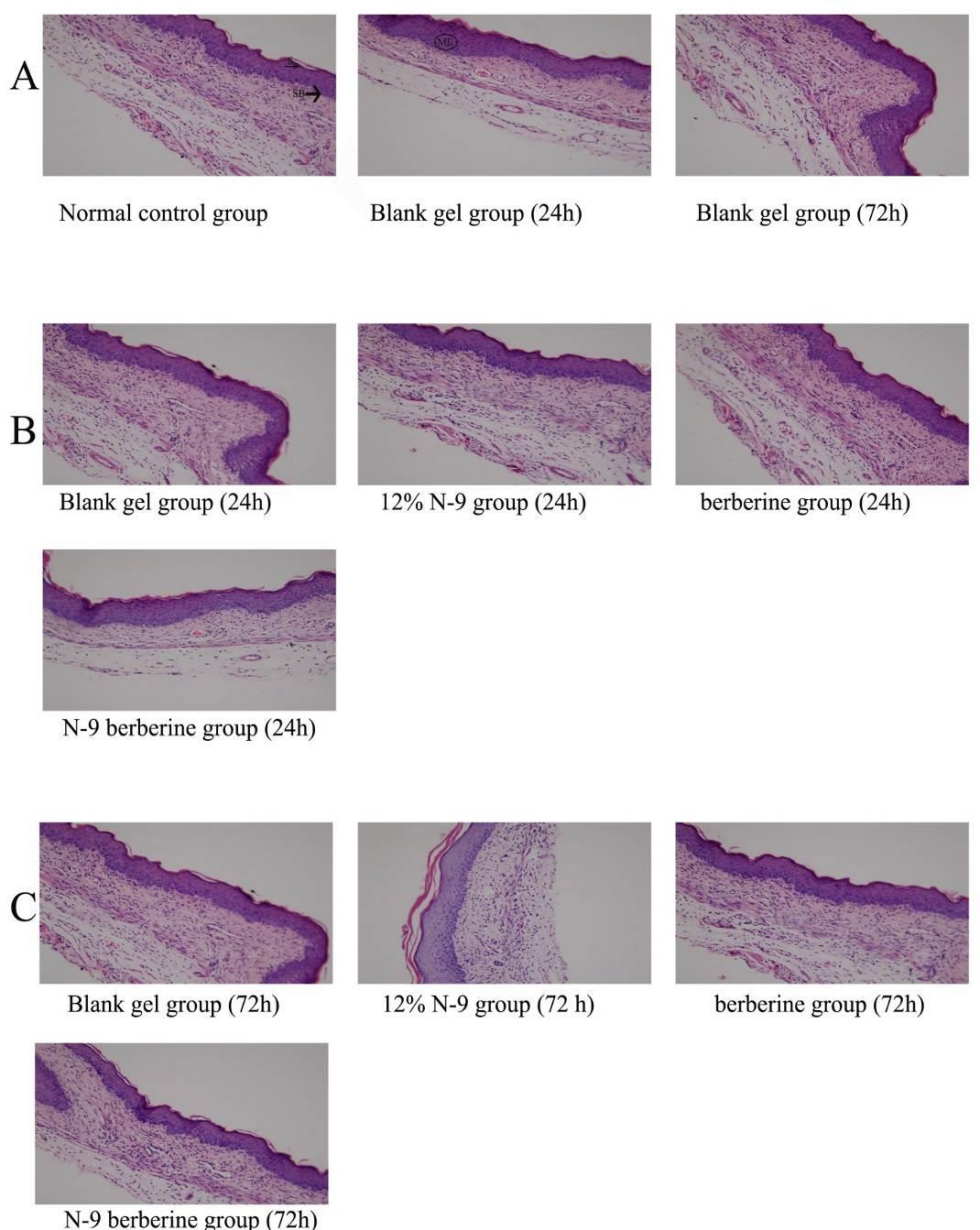
Histology of all observation groups’ vaginas under HE staining. (Magnification×200)

In all groups, surface middle lamella and stratum basale of mice vaginal stratified epithelium arranged regularly and equally; cell boundary was clear; structure was normal in both nucleus and cytoplasm; mucosa did not show inflammation (Figure 1). There was no obvious abnormality in morphological changes of the mice’s vaginas in 24h and 72h of observation group after treatment (Figure 1B, 1C). 

**SLPI** **,** **SP-D and LF protein expression level**

**Figure 2:**
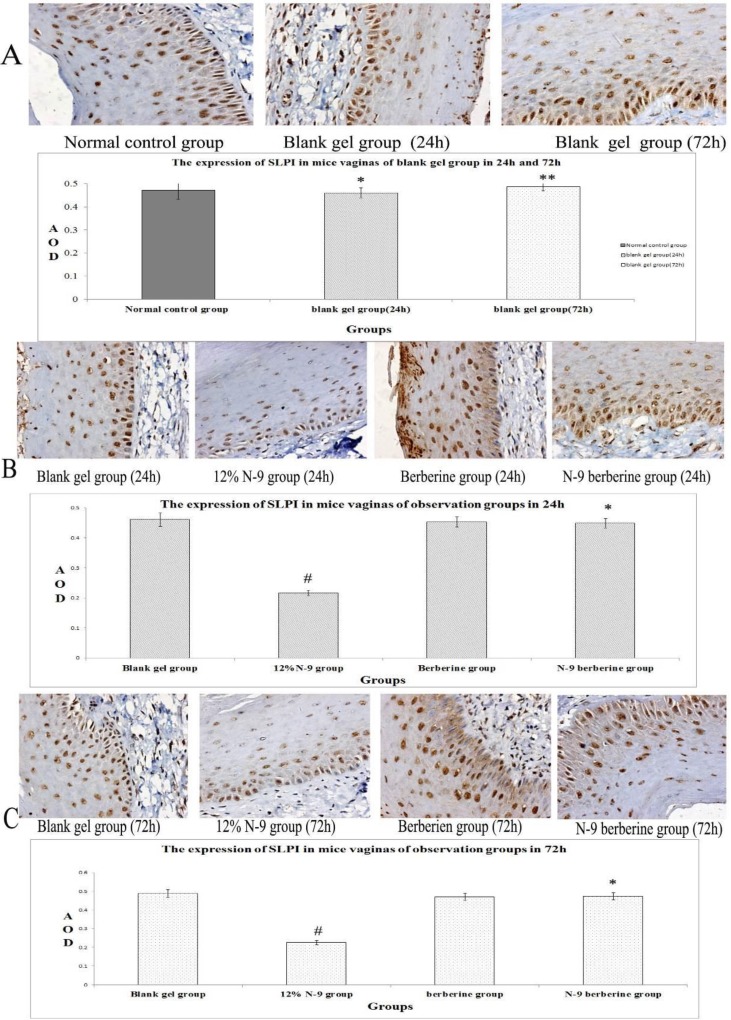
Immunohistochemical detection of SLPI-producing cells in mice’s vaginal tissue.

Immunohistochemistry staining results are shown in Figure 2, 4, 6. It was found that SLPI SP-D and LF of all observation groups expressed in the endochylema and cell membrane of vaginal epithelial tissues, presenting to be brown particles.

There was no statistical significance of positive expression level of the three indexes between normal control group and blank gel group in either 24h or 72h (p=0.420, 0.135 and 0.508 respectively in 24h, p=0.508, 0.383 and 0.788 respectively in 72h) (Figure 2A, Figure 4A, Figure 6A), while compared the expression of N-9 berberine plural gel group with blank gel group, the difference was without statistical significance (p=0.181, 0.533 and 0.258 respectively in 24h sub observation groups, p=0.863, 0.247 and 0.549 respectively in 72h sub observation groups) (Figure 2B, 2C; Figure 4B, 4C; Figure 6B, 6C). However, expression level of 12% N-9 gel group reduced compared to that of blank gel group (p=0.000 in both 24h and 72h sub observation groups) (Figure 2B, 2C; Figure 4B, 4C; Figure 6B, 6C). 

**Figure 3:**
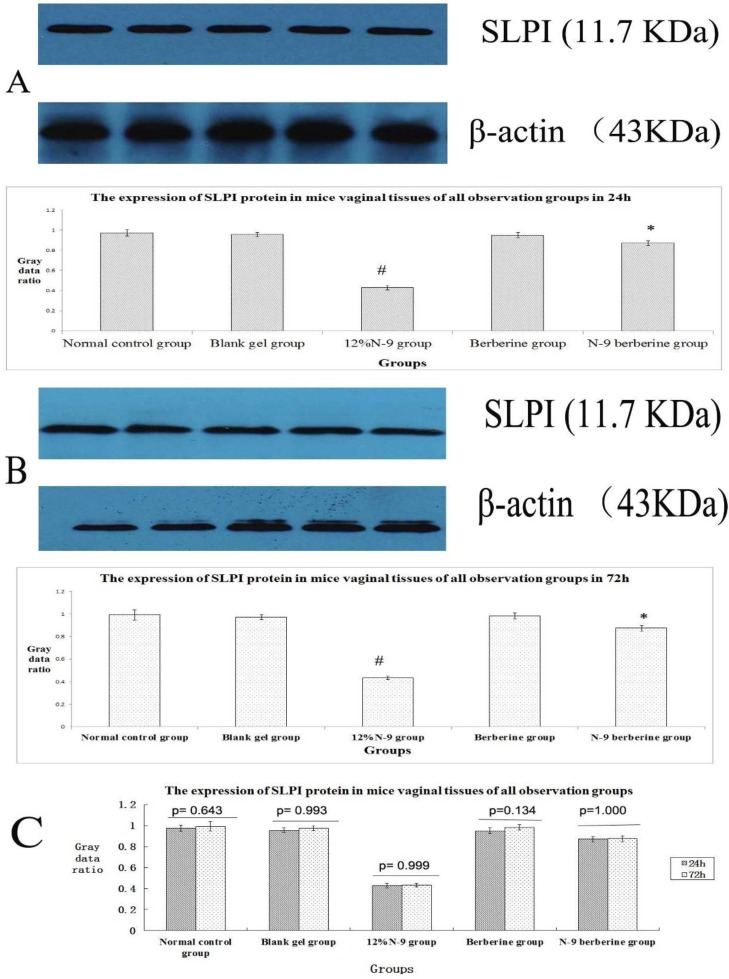
Western blotting of SLPI protein in mice’s vaginal tissue.

When Western blot method was used, target strips can be detected at 11.7 KDa, 47 KDa and 78 KDa (Figure 3，Figure 5, Figure 7）in all observation groups. The difference of three index was without statistical significance between normal control group and blank gel group in either 24h or 72h (p=0.367, 0.239 and 0.915 respectively in 24h, p= 0.278, 0.945 and 0.471 respectively in 72h) (Figure 3A，3B; Figure 5A, 5B; Figure 7A, 7B); the expression level of the three indexes in 12% N-9 gel group was decreased as compared with those in blank gel group (p=0.000, 0.002 and 0.000 respectively in 24h sub observation groups, p=0.000 in 72 sub observation groups) (Figure 3A，3B; Figure 5A, 5B; Figure 7A, 7B); there was no statistical significance in expression level of the three index between N-9 berberine plural gel group and blank gel group (p=0.185, 0.586 and 0.636 respectively in 24h sub observation groups, p=0.214,0.979 and 1.000 respectively in 72 sub observation groups), while the three protein expression level between N-9 berberine plural gel group and 12% N-9 group were of statistical significance (p= 0.000,0.004 and 0.000 respectively in 24h sub observation groups, p= 0.000,0.000 and 0.002 respectively in 72h sub observation groups) (Figure 3A，3B; Figure 5A, 5B; Figure 7A, 7B). 

Our findings also demonstrated that the expression of SLPI, SP-D and LF between 24h observation group after treatment and corresponding 72h observation group after treatment was also without statistical significance (p values are shown in Figure 3C, Figure 5C, Figure 7C respectively). The results above indicated that vaginal administration of N-9 berberine plural gel had little effect on the expression of SLPI SP-D and LF level in mice vaginas.

(A) mice vaginas in blank gel groups in vivo;

(B) mice vaginas in 24 sub observation groups in vivo;

(C) mice vaginas in 72 sub observation groups in vivo.

S: surface, ML: middle lamella, SB: stratum basale.

The endochylema and cell membrane of epithelial cells in mice’s vaginas are intensely stained.

(A) Representative immunohistochemical staining in blank gel group. Data about average optical density comparisons are shown in following bar chart (*,**p >0.05 versus normal control group).

(B) Representative immunohistochemical staining in 24 sub observation groups. Data about average optical density comparisons are shown in following bar chart (*p >0.05versus blank gel group; # p<0.05 versus blank gel group).

(C) Representative immunohistochemical staining in 72 sub observation groups. Data about average optical density comparisons are shown in following bar chart (*p >0.05 versus blank gel group ; # p <0.05 versus blank gel group).

Western blot method was detected SLPI protein of mice’s vaginal tissue and the protein was detected at 11.7KDa; each lane of

protein signal corresponded to one label in the abscissa axis of following bar chart.

(A) Representative western blotting of SLPI in 24 sub observation groups (above). Data about gray data ratio comparisons are

shown in the bar chart (below) (*p*>0.05, normal control group versus blank gel group; * p > 0.05 versus blank gel group; # *p *<0.05 versus blank gel group).

(B) Representative western blotting of SLPI in 72 sub observation groups (above). Data about gray data ratio comparisons are

shown in the bar chart (below). (*p*>0.05, normal control group versus blank gel group; * p > 0.05 versus blank gel group; # *p *<0.05 versus blank gel group)

(C) Data about gray data ratio comparisons between 24 and 72 sub observation groups are shown in the bar chart.

**Figure 4:**
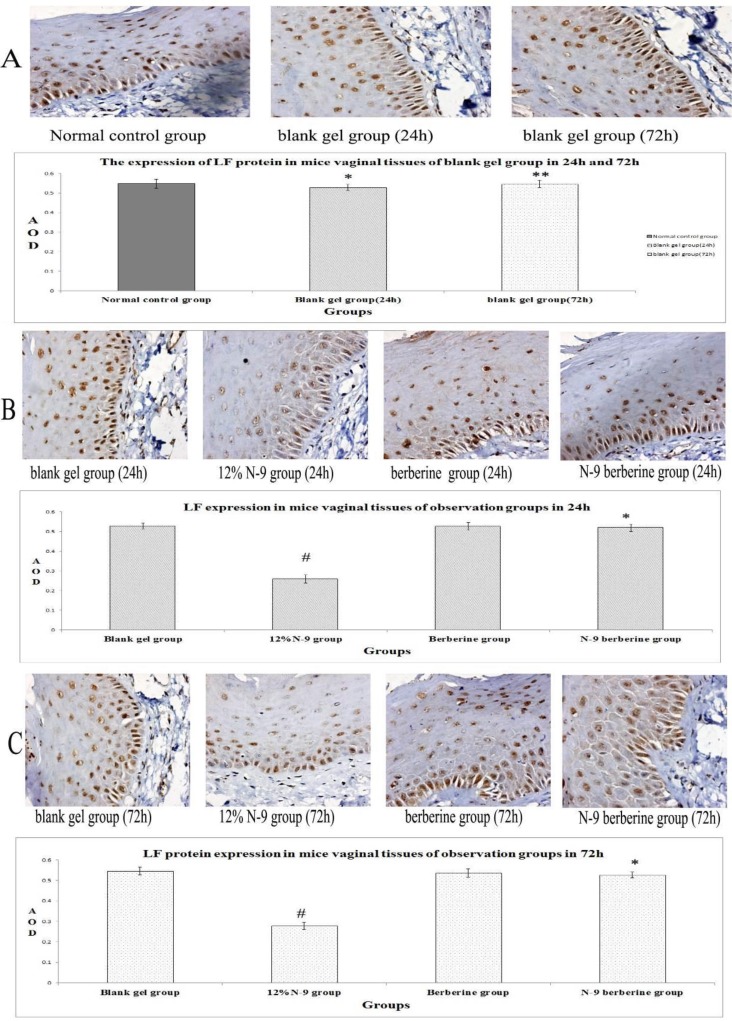
Immunohistochemical detection of LF-producing cells in mice’s vaginal tissue.

The endochylema and cell membrane of epithelial cells in mice’s vaginas are intensely stained.

(A) Representative immunohistochemical staining in blank gel group. Data about average optical density comparisons are shown in following bar chart (*,**p >0.05 versus normal control group).

(B) Representative immunohistochemical staining in 24 sub observation groups. Data about average optical density comparisons are shown in following bar chart (*p >0.05versus blank gel group; # p<0.05 versus blank gel group).

(C) Representative immunohistochemical staining in 72 sub observation groups. Data about average optical density comparisons are shown in following bar chart (*p >0.05 versus blank gel group; # p <0.05 versus blank gel group).

**Figure 5:**
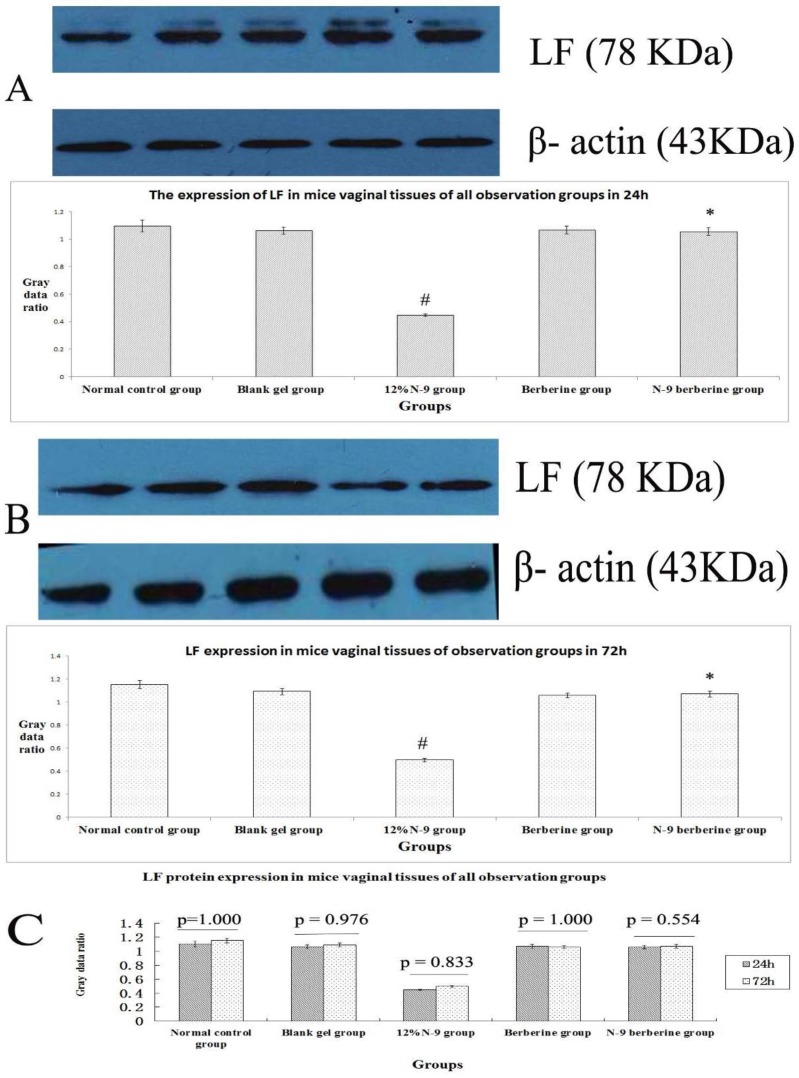
Western blotting of LF protein in mice’s vaginal tissue.

Western blot method was detected LF protein of mice’s vaginal tissue and the protein was detected at 78KDa; each lane of protein signal corresponded to one label in the abscissa axis of following bar chart.

(A) Representative western blotting of LF in 24 sub observation groups (above). Data about gray data ratio comparisons are shown in the bar chart (below) (*p*>0.05, normal control group versus blank gel group; * p > 0.05 versus blank gel group; # *p *<0.05 versus blank gel group).

(B) Representative western blotting of LF in 72 sub observation groups (above). Data about gray data ratio comparisons are shown in the bar chart (below). (*p*>0.05, normal control group versus blank gel group; * p > 0.05 versus blank gel group; # *p *<0.05 versus blank gel group)

(C) Data about gray data ratio comparisons between 24 and 72 sub observation groups are shown in the bar chart.

**Figure 6:**
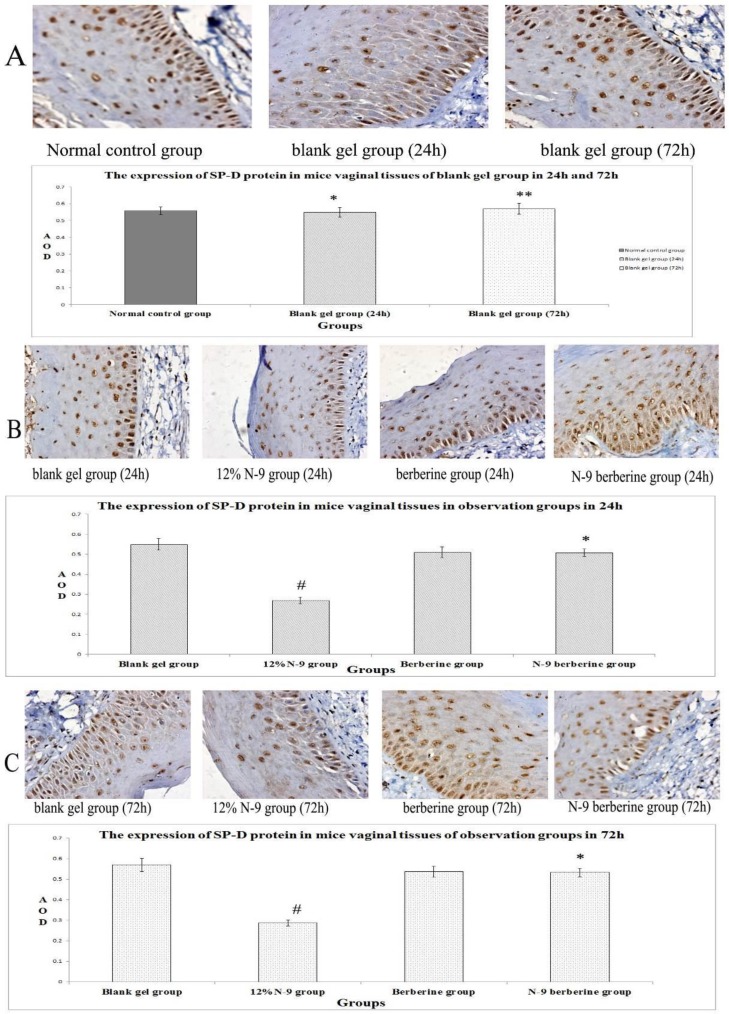
Immunohistochemical detection of SP-D-producing cells in mice’s vaginal tissue.

The endochylema and cell membrane of epithelial cells in mice’s vaginas are intensely stained.

(A) Representative immunohistochemical staining in blank gel group. Data about average optical density comparisons are shown in following bar chart (*,**p >0.05 versus normal control group).

(B) Representative immunohistochemical staining in 24 sub observation groups. Data about average optical density comparisons are shown in following bar chart (*p >0.05versus blank gel group; # p<0.05 versus blank gel group).

(C) Representative immunohistochemical staining in 72 sub observation groups. Data about average optical density comparisons are shown in following bar chart (*p >0.05 versus blank gel group; # p <0.05 versus blank gel group).

**Figure 7:**
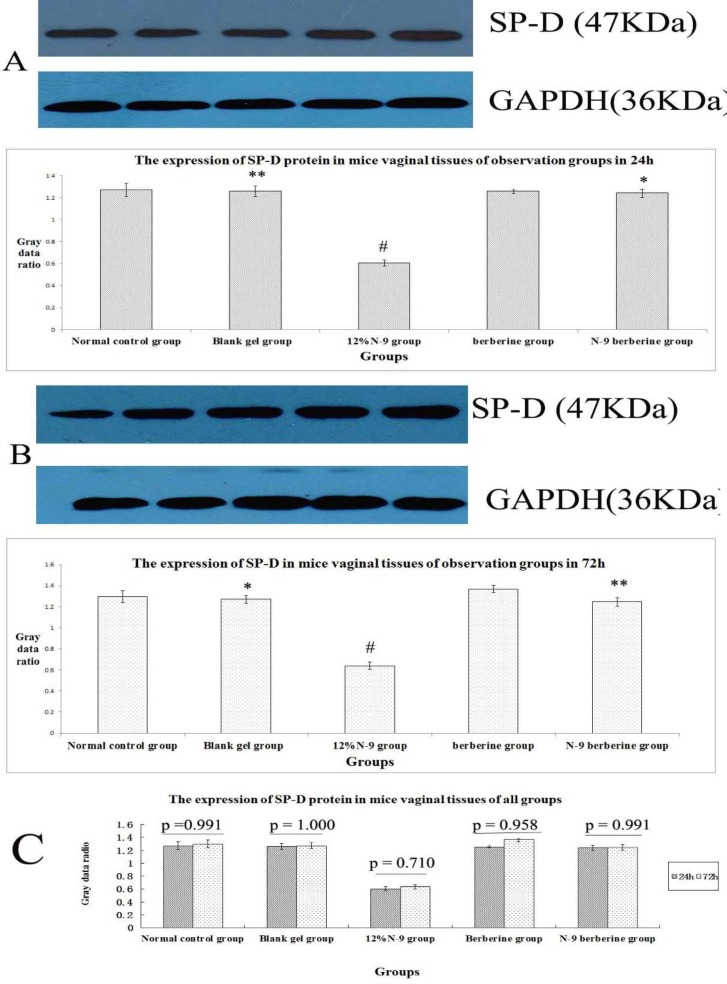
Western blotting of SP-D protein in mice’s vaginal tissue.

Western blot method was detected SP-D protein of mice’s vaginas in five groups and the protein was detected at 47 KDa; each lane of protein signal corresponded to one label in the abscissa axis of following bar chart.

(A) Representative western blotting of SP-D in 24 sub observation groups (above). Data about gray data ratio comparisons are shown in the bar chart (below) (p>0.05, normal control group versus blank gel group; * p > 0.05 versus blank gel group; # p <0.05 versus blank gel group).

(C) Representative western blotting of LF in 72 sub observation groups (above). Data about gray data ratio comparisons are shown in the bar chart (below). (p>0.05, normal control group versus blank gel group; * p > 0.05 versus blank gel group; # p <0.05 versus blank gel group)

(C) Data about gray data ratio comparisons between 24 and 72 sub observation groups are shown in the bar chart.

## Discussion

Berberine had ranges of anti-inflammatory and anti-bactericidal functions. Iwasa *et* *al* found that aromaticring C contained in its quaternary ammonium structure was necessary for antibiotic activity of Berberine (15-17). Berberine gel, the essential component of No.1 Jieze Gel, which has been used for curing cervical erosion and vaginitis for many years in our hospital, was curative. N-9 was a nonionic surfactant, being a killing-sperm drug of external application acknowledged internationally (18). However, its frequent use would dramatically damage cervicovaginal mucosal cells, increased the occurrence of vaginal lessions and elevated proinflammatory cytokines in cervicovaginal secretions, ultimately leading to the increased chance of infection. In addition, the side effect was positively correlated with the dose of N-9 (19-21). 

Our pre-study research found that N-9 berberine plural gel not only prevents *candida albicans vaginitis* and *trichomonas vaginitis*, but shows apparent effects of sperm-killing and antifertility on both rabbit and mice, which reduce the dose use of N-9 in clinic: the difference in antifertility incidence between N-9 berberine plural gel (10% N-9 contained) and 12% N-9 gel was without statistical significance (7, 8, 10, 11). In this study, we compared the influence of N-9 berberine plural gel with 12% N-9 gel on the vaginal antimicrobial peptides at 24h and 72h after treatment. Normal mice estrus was generally 8-24h, which was too short to grasp. Consequently each mouse was injected estrogen at physiological dose every other day to maintain mice estrus (13).

The mucosal surfaces of the human FRT were protected against pathogens by both the innate and the adaptive immunity. SLPI, SP-D and LF were important antimicrobials, which had a synergistic effect on innate immune in the FRT. The decrease of these may cause low defensiveness in genital tract, which could correlate with susceptibility to RTIs (22). Our results suggested that expression level of three indexes in 12% N-9 gel group were decreased, while vaginal N-9 berberine plural gel for one time had little influence on the expression of SLPI, LF and SP-D. SLPI expression in the human vaginas was decreased by certain pathogens, including *Trichomonas, Pseudomonas, Staphylococcus aureus, Chlamydia *and* Candida albicans* (23). 

Some scholars considered that it was related to the positive ion destroying the surface of bacterial cell membrane (24). Clinical trials had demonstrated the high efficiency of LF against infections in inflammatory diseases, such as bacteria, viruses and fungi. The antimicrobial mechanisms of LF were considered to be at least three aspects: it band iron molecules with high afﬁnity, which inhibited bacterial growth by sequestering free iron under acidic conditions; it augmented the permeability of bacterial cell membrane, leading to LPS liberation from tunica externa; it produced antimicrobial peptides and antibacterial action via hydrolyzation (25, 26). 

SP-D was a candidate molecule to prevent vaginal infection in vaginal mucosal surfaces. SP-D recognized non-self-carbohydrates and lipid moieties on the surface of bacterial, fungal and viral pathogens, and then mediated their elimination (27, 28). It enhanced the uptake of pathogens through different mechanisms, including opsonizing the pathogens, activating ligands and regulating cell-surface-receptor expression (29).

## Conclusion

In conclusion, N-9 berberine plural gel demonstrated broad-spectrum activity in the previous tested animal models (9, 30, 31). These results, combined with the little influence of it on the antimicrobial peptides in normal mice vaginas, suggested that N-9 berberine plural gel may prove to be useful as a vaginal spermicidal in human, and that clinical evaluation of its spermicidal and microbicidal activities were warranted.
